# Perceptions of Independent Pharmacist Prescribing among Health Authority- and Community-Based Pharmacists in Northern British Columbia

**DOI:** 10.3390/pharmacy9020092

**Published:** 2021-04-23

**Authors:** Jordan Lewis, Arden R. Barry, Katie Bellefeuille, Robert T. Pammett

**Affiliations:** 1 GR Baker Memorial Hospital, Northern Health Authority, 543 Front Street, Quesnel, BC V2J 2K7, Canada; jordan.lewis3@northernhealth.ca; 2 Chilliwack General Hospital, Lower Mainland Pharmacy Services, 45600 Menholm Rd, Chilliwack, BC V2P 1P7, Canada; arden.barry@ubc.ca; 3 University Hospital of Northern British Columbia, Northern Health Authority, 1475 Edmonton Street, Prince George, BC V2M 1S2, Canada; katie.bellefeuille@northernhealth.ca; 4 Pharmacy Services, Northern Health Authority, 404-299 Victoria St., Prince George, BC V2L 5B8, Canada

**Keywords:** pharmacists, pharmacy, drug prescriptions, health services, pharmacy research

## Abstract

Pharmacists across Canada have varying degrees of ability to prescribe medications depending on their jurisdiction of licensure. The purpose of this study was to the evaluate attitudes, beliefs, and perceptions of independent pharmacist prescribing among health authority- and community-based pharmacists. This prospective, cross-sectional online survey assessed the perceptions of independent pharmacist prescribing of health authority and community pharmacists practising in northern British Columbia (BC), which was defined as within the geographical boundaries of Northern Health. Responses were analysed using descriptive statistics and a regression analysis. There were 45 respondents to the survey: 22 community pharmacists and 23 health authority pharmacists. Both community and health authority pharmacists held positive perceptions of independent pharmacist prescribing and did not identify any barriers to incorporating independent pharmacist prescribing into their practice. Respondents were highly likely to apply for independent pharmacist prescribing authority, if available. Pharmacists in BC are currently not able to independently prescribe schedule I medications. The provincial regulatory body has proposed a framework for a Certified Pharmacist Prescriber designation, which if approved would allow pharmacists to prescribe in collaborative practice settings. This study provides some insight into the perception of pharmacists in northern BC in pursuing this designation, which may be valuable for planning purposes in case of adoption of the framework. These results are also likely applicable to other non-urban practice settings in Canada. Pharmacists in northern BC perceived independent pharmacist prescribing positively and a high proportion were likely to apply for this authority if it were permitted via legislation.

## 1. Introduction

It is well recognised that pharmacists have the skills and knowledge to benefit patients beyond the dispensation of medications [1,2,3,4,5,6,7,8,9,10,11,12,13,14,15,16,17,18,19,20]. As such, the scope of practice of pharmacists has evolved to provide better care to patients across Canada and internationally. One such example is the prescribing of schedule I (prescription only) medications by pharmacists. In Canada, only pharmacists in the province of Alberta have the authority to prescribe schedule I drugs independently (i.e., without the authorisation of another regulated health professional such as a physician, nurse practitioner, or dentist) [1]. Outside of Alberta, the authority to prescribe among pharmacists varies from province to province [1]. Independent prescribing authority has been available to pharmacists in the United Kingdom since 2006 and in New Zealand since 2013 [2,3]. In the United States, designated pharmacists have the authority to prescribe medications in collaborative care settings with other prescribers [4,5,6,7,8,9,10,11,12,13,14].

Evidence has demonstrated that independent pharmacist prescribing in the United States results in improved outcomes for patients with certain medical conditions (specifically stroke, cancer, and chronic pain), which has been associated with reduced costs [4,5]. Since its introduction in 2007, pharmacist prescribing in Alberta, Canada has been associated with improved health outcomes, including improved control of hypertension, diabetes, and dyslipidemia [15,16,17]. Additionally, pharmacist prescribing has been shown to reduce overall cardiovascular risk, as well as reduce the cost of care [18,19]. Patients who received care from pharmacist prescribers have reported a positive experience and supported further expansion of pharmacist prescribing authority [20].

Patients in non-urban areas of Canada have less access to health care providers than patients in urban areas [21]. This may lead pharmacists in such areas to desire to practice with extended clinical scope to better meet the needs of their patients. A recent study carried out in North Carolina found that non-urban pharmacists were likely to report desire for expanded clinical services [22]. Thus, non-urban health authority pharmacists may be more likely to hold positive perceptions of independent pharmacist prescribing compared to their urban counterparts.

Pharmacists in the province of British Columbia, Canada are currently not able to independently prescribe schedule I medications. Recently, the provincial regulatory body (the College of Pharmacists of British Columbia, Vancouver, BC, Canada) proposed a framework for a Certified Pharmacist Prescriber designation [23]. If approved, this designation would allow for pharmacists to prescribe in collaborative practice settings (currently described as “a relationship with a regulated health professional who has the authority to prescribe to: facilitate communication, determine mutual goals of therapy that are acceptable to the patient, share relevant health information and establish the expectations of each regulated health professional when working with a mutual patient.”) [23].

The objective of this study was to determine the perceptions of, attitudes toward, and beliefs about independent pharmacist prescribing among health authority (those working in government-funded public healthcare settings)—and community-based (those working in private, for-profit pharmacies) pharmacists in northern British Columbia, as well as to identify potential barriers and enablers to the implementation of independent pharmacist prescribing and assess respondents’ intention to apply for prescribing authority if it were to become available.

## 2. Methods

### 2.1. Study Design

This prospective, observational, online survey study assessed the perceptions of pharmacists in northern British Columbia, Canada about independent pharmacist prescribing. The survey questions were modified from previous similar surveys and are available from the corresponding author on request [24,25,26]. All Northern Health (i.e., the government-funded public health authority in northern British Columbia) pharmacists received an e-mail invitation to participate in the survey. Furthermore, all pharmacies within the boundary of Northern Health were faxed an invitation to participate in the survey. The Northern Health region is comprised of over 24 communities in the northern half of British Columbia, which covers over 617,000 km^2^. The survey was anonymous, and questions were designed to limit the risk of respondents being identified based on their responses, as some communities within Northern Health have a limited number of pharmacists.

### 2.2. Data Collection

The online survey link was distributed to participants via an e-mail distribution list for 67 Northern Health pharmacists and via facsimile to community pharmacies. The denominator for community pharmacists was estimated based on the number of pharmacists in the northern British Columbia communities, as per the provincial regulatory body (i.e., College of Pharmacists of British Columbia, Vancouver, BC, Canada) database of workplace location. Informed consent was obtained from participants prior to responding to the survey. The survey was open for four weeks (23 January to 20 February 2020) and participants were sent one reminder after two weeks.

### 2.3. Statistical Analysis

Data was analysed with Microsoft Excel (version 12, Microsoft Corporation, Redmond, WA, USA) using descriptive statistics. A regression analysis was performed using SPSS (IBM SPSS Statistics for Windows, version 24.0, IBM Corporation, Armonk, NY, USA) to identify potential demographic characteristics that may have been associated with the likelihood to apply for prescribing authority.

## 3. Results

A total of 45 pharmacists responded to the survey. Twenty-two of the respondents were community pharmacists (estimated to be a response rate of 11%) and 23 were health authority pharmacists (response rate of 34%). Respondent demographic characteristics are included in Table 1. The groups did not differ significantly in characteristics except for their level of education.

**Figure 1:**
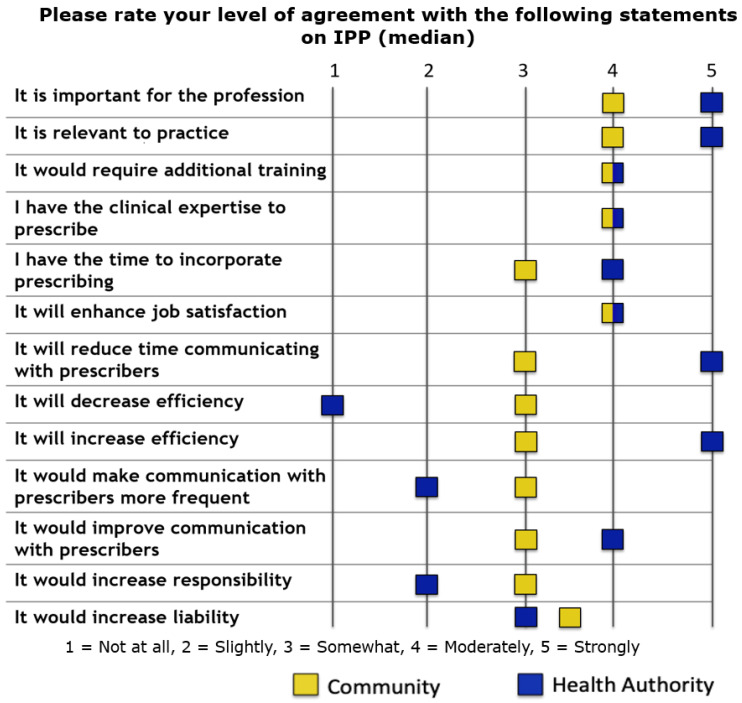
Respondents’ attitudes and beliefs about independent pharmacist prescribing. * One question (not displayed in figure above) had a sample of 18 rather than 45 due to survey error.

Figure 1 includes respondents’ attitudes towards and beliefs about independent pharmacist prescribing. There was a high degree of at least moderate agreement among both health authority- and community-based pharmacists regarding positive statements about independent pharmacist prescribing. Health authority-based pharmacists generally responded with higher levels of agreement to positive statements about independent pharmacist prescribing (e.g., it is important for the profession, relevant to practice, and would increase efficiency) versus community-based pharmacists.

**Figure 2:**
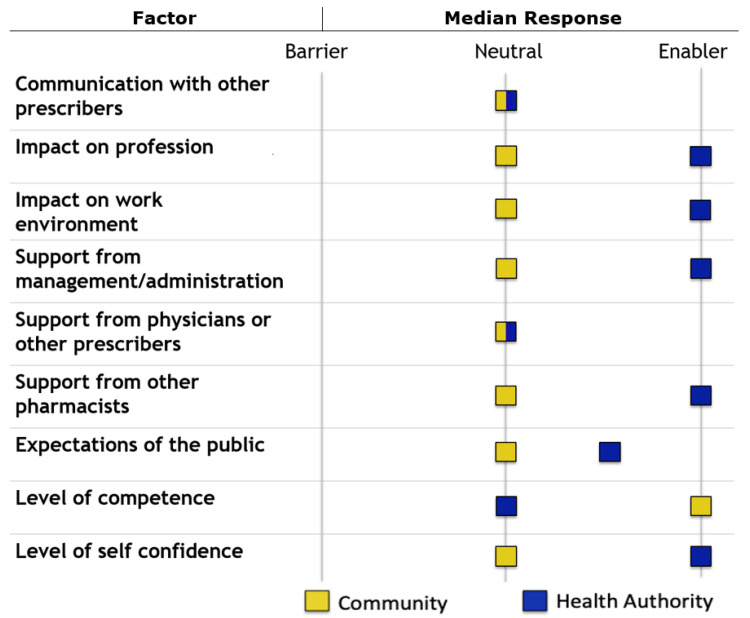
Respondents’ perceived enablers and barriers to incorporating independent pharmacist prescribing into their practice.

**Figure 3:**
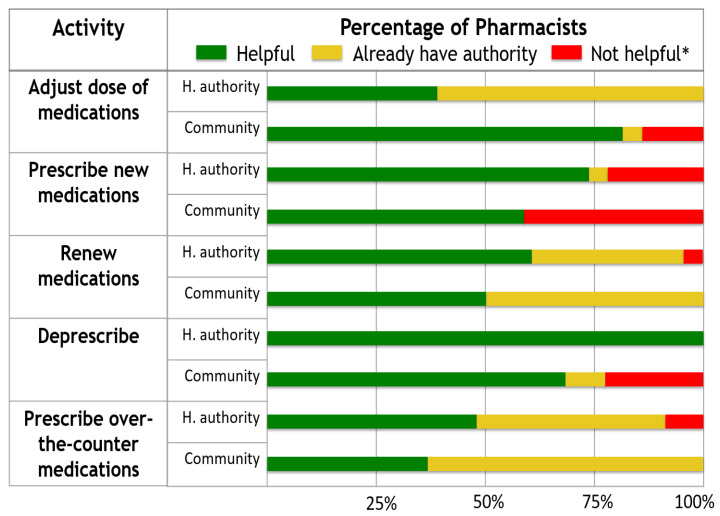
Respondents’ perceived effects of independent pharmacist prescribing on workplace activities. * “Not helpful” is a combination of the following responses: Not applicable, do not envision myself doing this, not relevant, or do not have the skill. Abbreviations: H. authority = health authority.

Survey respondents identified no perceived barriers to incorporating independent pharmacist prescribing into their respective practices (Figure 2). Health authority-based pharmacists were more likely to rate that independent pharmacist prescribing would be helpful for the pre-populated list of practice activities, or that they already had the authority to perform these activities (Figure 3).

When assessing the intentions of northern British Columbia pharmacists to apply for prescribing authority, it was found that 89% of respondents overall would be likely to apply for prescribing authority if made available. The regression analysis did not demonstrate any statistically significant associations between respondent characteristics’ and likelihood of applying for independent pharmacist prescribing. Eighty-seven percent of respondents were likely to apply for independent pharmacist prescribing authority if additional training was required, and there were no statistically significant differences between responses of health authority- and community-based pharmacists. Most pharmacists were willing to pay at least $1000 CAD to obtain prescribing authority, with health authority pharmacists willing to pay a higher mean amount than community pharmacists.

## 4. Discussion

The online survey of pharmacists’ perceptions of independent pharmacist prescribing had an estimated response rate of 17% among pharmacists in northern British Columbia. A higher response rate among health authority-based pharmacists (34%) versus community-based pharmacists (estimated at 11%) was expected, as this group may have had more interest in collaborative prescribing. Health authority-based pharmacists may also have been more likely to participate due to receiving the invitation via e-mail versus facsimile due to the convenience of accessing an online survey. With the exception of education level, there were no statistically significant differences between health authority- and community-based pharmacists with respect to demographic characteristics. Even though hospital pharmacists had a higher level of pharmacy education, the overall results were similar between groups. As such, the desire to expand scope of practice does not seem to be dependent on level of education. Despite the differences in practice settings, respondents were highly likely overall to report working in a collaborative environment with other prescribers. Therefore, many community pharmacists perceived themselves to practice in a collaborative practice setting despite likely not being located in the same physical location as the other prescribers.

A study by Prasad and colleagues was the first study to assess health authority pharmacists’ perceptions of independent pharmacist prescribing in British Columbia. The study was conducted across mostly urban settings in the Lower Mainland of British Columbia, and it found that pharmacists held mostly positive perceptions of independent pharmacist prescribing and would be likely to apply for the authority, if available. However, it did not include pharmacists practicing in community-based settings.

Community-based pharmacists were more likely to somewhat or moderately agree with positive statements about independent pharmacist prescribing, whereas health authority-based pharmacists were more likely to strongly agree with these statements. This may be because the framework for pharmacist prescribing developed by the College of Pharmacists of British Columbia is potentially more relevant to hospital-based practice. Pharmacists in this study did not identify any barriers to incorporating independent pharmacist prescribing into their practices. However, health authority-based pharmacists were more likely to identify factors as enablers, whereas community-based pharmacists were more likely to identify factors as being neutral. Similar to the findings regarding beliefs about independent pharmacist prescribing, this may be because collaborative prescribing is more relatable to hospital practice.

Pharmacists in northern British Columbia were more likely to identify independent pharmacist prescribing to be helpful for practice-based activities, or that they already had the authority to prescribe in these circumstances. These results suggest that respondents generally have positive perceptions about the impact of independent pharmacist prescribing on their practice environments. When compared to the survey of urban pharmacists from Vancouver, British Columbia and the surrounding area, pharmacists in northern British Columbia were more likely to identify as already having authority for prescribing activities [24]. This may be due to fewer health care providers in northern British Columbia; accordingly, more northern pharmacists may be required to perform prescribing activities than pharmacists in Vancouver and the surrounding area.

A majority of respondents intend to apply for independent pharmacist prescribing if it were to be granted through legislation, which was similar among both health authority- and community-based pharmacists. Overall, this was higher among pharmacists in northern British Columbia, as compared to pharmacists in Vancouver (89% versus 66%, respectively) [24]. Again, this may indicate that pharmacists in the north of the province may have a greater perceived need to prescribe due to fewer health care providers with prescribing privileges. In addition, as the study by Prasad and colleagues was conducted in 2017, it may be reflective of a change in perception, as pharmacists continue to advocate for an expanded scope of practice.

With respect to limitations, this survey had a relatively small sample size and therefore is at risk of sampling and response bias. In addition, as the survey was conducted prior to the implementation of independent pharmacist prescribing, it should be noted that respondents’ perceptions, attitudes, and beliefs might differ once this authority is actually granted. Therefore, future studies are warranted to assess pharmacists’ beliefs regarding independent prescribing once it is implemented in practice.

## 5. Conclusions

Pharmacists in northern British Columbia demonstrated generally positive perceptions of independent pharmacist prescribing. Despite differences between health authority- and community-based pharmacists with respect to attitudes towards and beliefs about independent pharmacist prescribing, a high proportion of respondents in both groups would be likely to apply for this authority if it were to be granted.

## Figures and Tables

**Table 1:** Survey respondents characteristics (*n* = 45).

Characteristic	Community (*n* = 22)	Health Authority (*n* = 23)	*p*-Value
Female	36.4%	56.5%	0.17
Age < 40 years	59.1%	77.2%	0.17
>5 years of pharmacist experience	63.6%	65.2%	0.91
Location within Prince George	50%	73.9%	0.10
Staff pharmacist role	63.6%	65.2%	0.73
Perceived collaborative relationship *	90.9%	95.7%	0.54
Highest Level of Pharmacy-Related Education			
Entry-level pharmacy degree	86.4%	26%	<0.001
Beyond entry-level pharmacy degree	5%	74%	<0.001

* defined as “a relationship with a regulated health professional who has the authority to prescribe to: facilitate communication, determine mutual goals of therapy that are acceptable to the patient, share relevant health information and establish the expectations of each regulated health professional when working with a mutual patient” [23].

## Data Availability

Aggregate data presented in this study are available on request from the corresponding author.
